# Prevalence of polypharmacy and potentially inappropriate medication use in older lung cancer patients: A systematic review and meta-analysis

**DOI:** 10.3389/fphar.2022.1044885

**Published:** 2022-12-16

**Authors:** Fangyuan Tian, Zhaoyan Chen, Dan Zhou, Li Mo

**Affiliations:** ^1^ Department of Pharmacy, National Clinical Research Center for Geriatrics, West China Hospital of Sichuan University, Chengdu, China; ^2^ Department of Epidemiology and Health Statistics, West China School of Public Health and West China Fourth Hospital, Sichuan University, Chengdu, China; ^3^ The Center of Gerontology and Geriatrics, National Clinical Research Center of Geriatrics, West China Hospital of Sichuan University, Chengdu, Sichuan, China

**Keywords:** polypharmacy, potentially inappropriate medication, older people, lung cancer, meta-analysis

## Abstract

**Objectives:** In older lung cancer patients, polypharmacy and the use of potentially inappropriate medications (PIMs) are commonly reported, but no systematic review or meta-analysis has been carried out to ascertain the prevalence and risk variables in this group. This study aimed to identify the prevalence of polypharmacy, PIMs and associated risk variables in older lung cancer patients.

**Methods:** We searched for articles from the beginning to February 2022 in PubMed, Embase, and Web of Science that related the use of PIMs and polypharmacy by older lung cancer patients (PROSPERO Code No: CRD42022311603). Meta-analysis was performed on observational studies describing the prevalence and correlation of polypharmacy or PIMs in older patients with lung cancer.

**Results:** Of the 387 citations, 6 articles involving 16,890 patients were included in the final sample. In older lung cancer patients pooled by meta-analysis, 38% and 35% of PIMs and polypharmacy, respectively. The prevalence of PIMs was 43%, 49%, and 28%, respectively, according to the 2019 AGS Beers criteria, 2014 screening tool for older people’s prescriptions/screening tool for alerting to the proper therapy (STOPP/START criteria) criteria, and other criteria.

**Conclusion:** This systematic review and meta-analysis demonstrated a high prevalence of polypharmacy and PIMs among older lung cancer patients. Therefore, it is essential to take rational interventions for older lung cancer patients to receive reasonable pharmacotherapy.

**Systematic Review Registration**: [https://www.crd.york.ac.uk/PROSPERO/], identifier [CRD42022311603].

## Introduction

With over 1.8 million deaths from lung cancer in 2020 and 18% of all deaths from cancer, lung cancer is the leading cause of cancer fatalities worldwide (Global Cancer Observatory, 2020; World Health Organization, 2022). Cancer development is associated with older age and is influenced by biological factors, including DNA damage and telomere shortening over time. In the upcoming years, it is anticipated that the incidence of lung cancer in older individuals will increase even more as the population ages (Decoster and Schallier, 2019). In approximately 37% of cases, the patient is over 75 years old. Men and women with lung cancer generally present at a median age of 70 years. Men are more likely to develop lung cancer at a rate of 585.9 per 100,000 in their 85–89 years of age, but women are more likely to develop lung cancer at a rate of 365.8 per 100,000 in their 75–79 years of age (Torre et al., 2016).

Most senior lung cancer patients always have coexisting chronic diseases, which adds to the burden of having to take multiple medications (Grose et al., 2014; Nilsson et al., 2017; Ding et al., 2020). Older individuals, however, may be more susceptible to changes in pharmacokinetics and pharmacodynamics due to aging, which could increase the likelihood of medication interactions and drug-disease interactions (Fried et al., 2014; Payne, 2016). Because chemotherapy may increase the likelihood of medication-drug interactions and adverse drug reactions (ADRs), which may include chemotherapy-related toxicity, cancer patients are particularly vulnerable to unintended effects of adverse drug events (Maggiore et al., 2014; Woopen et al., 2016). According to some research, older cancer patients were more likely to suffer comorbid conditions, geriatric syndrome, and frailty, which increased the incidence of polypharmacy and PIMs [(Wildiers et al., 2014), (Koczwara et al., 2022), (LeBlanc et al., 2015)].

A growing body of evidence shows that polypharmacy can have detrimental effects. Polypharmacy is defined as the usage of more than five drugs. It is associated with the prescription of unsuitable medications (Field et al., 2001; Ferner and Aronson, 2006; Maddison et al., 2011; Weng et al., 2013). Potentially inappropriate medications (PIMs) are those that should not be used in older individuals due to the high risk of adverse drug responses and/or lack of adequate evidence of benefits when safer, equivalent, or more effective treatment options are available (Tian et al., 2021). As the first expert consensus on geriatric PIMs, the AGS Beers criteria are regularly updated and reviewed by the American Geriatrics Society, which are now in their sixth iteration (Beers et al., 1991; American Geriatrics Society AGS Beers Criteria^®^ Update Expert Panel, 2019). The screening tool for older people’s prescriptions/screening tool for alerting to the proper therapy (STOPP/START criteria) was created at the University College Cork using a Delphi methodology, and the second edition was revised in 2014 (O'Mahony et al., 2015; O'Mahony, 2020). These two standards have been applied widely in drug application surveys in communities, clinics, and hospitals around the world.

These criteria have also been used in certain studies to investigate the prevalence of polypharmacy or PIMs among older lung cancer patients. To date, there have been no systematic reviews or meta-analyses about the use of PIMs and polypharmacy in older lung cancer patients. To overcome the shortcomings of past findings, we conducted the first systematic review on the prevalence of polypharmacy and PIMs in older lung cancer patients to provide pertinent evidence. The purpose of this meta-analysis is to increase sample size and indicate the direction for further research.

## Methods

### Search strategy

This study was carried out in accordance with the Preferred Reporting Items for Systematic Reviews and Meta-analysis Guidelines (Moher et al., 2015). This systematic review and meta-analysis was submitted to PROSPERO (CRD42022311603). We searched PubMed, Embase and Web of Science from inception to February 2022. For PubMed, the search items included (“Polypharmacy” [MeSH Terms] OR (“Potentially Inappropriate Medication” [Title/Abstract]) OR (“Potentially Inappropriate Prescription” [Title/Abstract]) OR (“Inappropriate Medication” [Title/Abstract]) OR (“Inappropriate Prescription” [Title/Abstract]) OR (“Inappropriate Prescribing” [Title/Abstract]) OR (“Inappropriate Drug Use” [Title/Abstract]) and (“Lung cancer” [Title/Abstract]). The prevalence of polypharmacy or PIMs was reported using any defined criteria in observational studies on older lung cancer patients who were published in the English language. Terms from both medical and non-medical topic headings were utilized in the search string. Additionally, to identify any potential studies, references to pertinent papers and reviews were made.

### Selection criteria and data extraction

The studies satisfied the following criteria (World Health Organization, 2022): Observational study design (Global Cancer Observatory, 2020). The reported prevalence of PIMs or polypharmacy in older lung cancer patients (Decoster and Schallier, 2019). Medication use assessed using any PIMs-specific stated criteria (Torre et al., 2016). Documentation of any elements that increase the likelihood that older people would use PIMs or polypharmacy. Studies were excluded if they (World Health Organization, 2022) did not include older lung cancer populations or (Global Cancer Observatory, 2020) did not describe the prevalence of polypharmacy and PIMs usage in the older lung cancer population and (Decoster and Schallier, 2019) were duplicate studies, reviews, case reports, interventional studies, and meta-analyses.

### Selection of studies

To ascertain whether each study complied with the predetermined inclusion criteria, two reviewers (FT and ZC) independently read the titles and abstracts of the studies. To determine whether further review was necessary, all 387 titles and abstracts were evaluated. The first 50 references were separately evaluated for quality control by a senior researcher (LM). The degree of agreement was 90%, with five inconsistencies that were discussed among the three reviewers to reach agreement. The two reviewer groups then conducted a second round of review on the remaining studies. The references of the retrieved articles were further searched in an effort to locate more appropriate articles.

### Quality assessment

Utilizing cross-sectional data from the Agency for Healthcare Research and Quality (AHRQ), the methodological quality of the included studies was assessed (Hu et al., 2015). The AHRQ evaluates the representativeness of the information source, inclusion and exclusion criteria, the time period, whether subjects were sequential, whether assessors of the subjective portions of the research were masked to other aspects of the participants’ status, any evaluations made for quality assurance objectives, any patient exclusions from the analysis, how confounding was examined, how to deal with missing data in the analysis, summarize the degree of response of patients and the integrity of data collection, and clarify follow-up measures. AHRQ scores range from 0 (lowest level) to 11 (highest level). Research with a score of 8 or more was regarded as high quality, while research with a score of less than 4 was regarded as low quality.

### Statistical analysis

STATA software was used to conduct the statistical analysis. The combined prevalence was expressed as percentages with a 95% confidence interval (95% CI), considering the population’s various real effect sizes. The random-effects model proposed by Der Simonian and Laird was used. To ascertain the relationship between different patient characteristics and the risk of polypharmacy and the use of PIMs, the pooled relative ratio was determined for each study.

## Results

### Basic information of the studies

**FIGURE 1:**
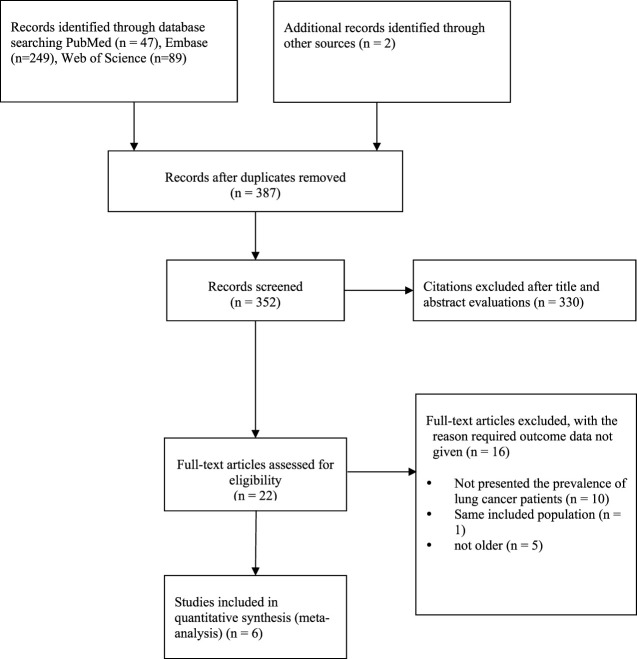
Diagram of the literature selection.

Through the use of resources such as PubMed, Embase, Web of Science, and others, 387 records in total were found. Using Endnote, 35 duplicates were eliminated. A total of 330 records were culled after title and abstract screening, leaving 22 full-text articles for additional analysis. Because the patients in ten of the papers were not lung cancer patients, the included population was the same across all trials, and the population was not old, these papers were not included in the analysis. Six papers (Lund et al., 2018; Hakozaki et al., 2020; Hakozaki et al., 2021; Mohamed et al., 2021; Ham et al., 2022; Tian et al., 2022) that satisfied the inclusion criteria were finally added to the study (Figure 1).

Table 1 provides a summary of the features of the included studies. These 6 studies used an observational study design and included 16,890 patients in total, representing the older lung cancer population. Four studies involved inpatients, and two studies involved outpatients. One study was undertaken in Europe, two studies were undertaken in the United States, and three studies were undertaken in Asia. Three studies used the AGS Beers criteria, two studies used the STOPP/START criteria, and one study used the criteria for patients with cancer in the palliative phase. With reference to the AHRQ, the studies received an average score of 7, which denoted moderate quality.

**TABLE 1:** Characteristics of included studies.

Article	Country	Study design	Tumor type	Sample size	Setting	Male (%)	PIMs criteria applied	Prevalence (%)		Quality of studies
								Polypharmacy	PIMs	
Tian et al., 2022 [20]	China	Cross-sectional	Lung cancer	1,275	Outpatients	56.55	2019 AGS Beers criteria	17.88	42.67	7
Hakozaki et al., 2021 [21]	Japan	Retrospective study	Advanced lung cancer	232	Inpatients	25.86	STOPP/START V2	38.4	31.9	7
Ham et al., 2021 [22]	Netherland	Observational study	Lung cancer at the end of life	7,864	Outpatients	67.43	OncPal Deprescribing Guideline	54.82	28.41	7
Mohamed et al., 2021 [23]	America	Cross-sectional	Lung cancer with physical functional impairments	125	Inpatients	—	2015 AGS Beers criteria	48	—	7
Hakozaki et al., 2020 [24]	Japan	Cross-sectional	Advanced non-small-cell Lung cancer	157	Inpatients	63.69	STOPP/START V2	59.87	38.22	7
Lund et al., 2018 [25]	America	Cross-sectional	I–II Lung cancer	7,237	Inpatients	42.48	2012 AGS Beers criteria	33.30	37–45	7

### Prevalence of polypharmacy and PIMs in the older Chinese population

**FIGURE 2:**
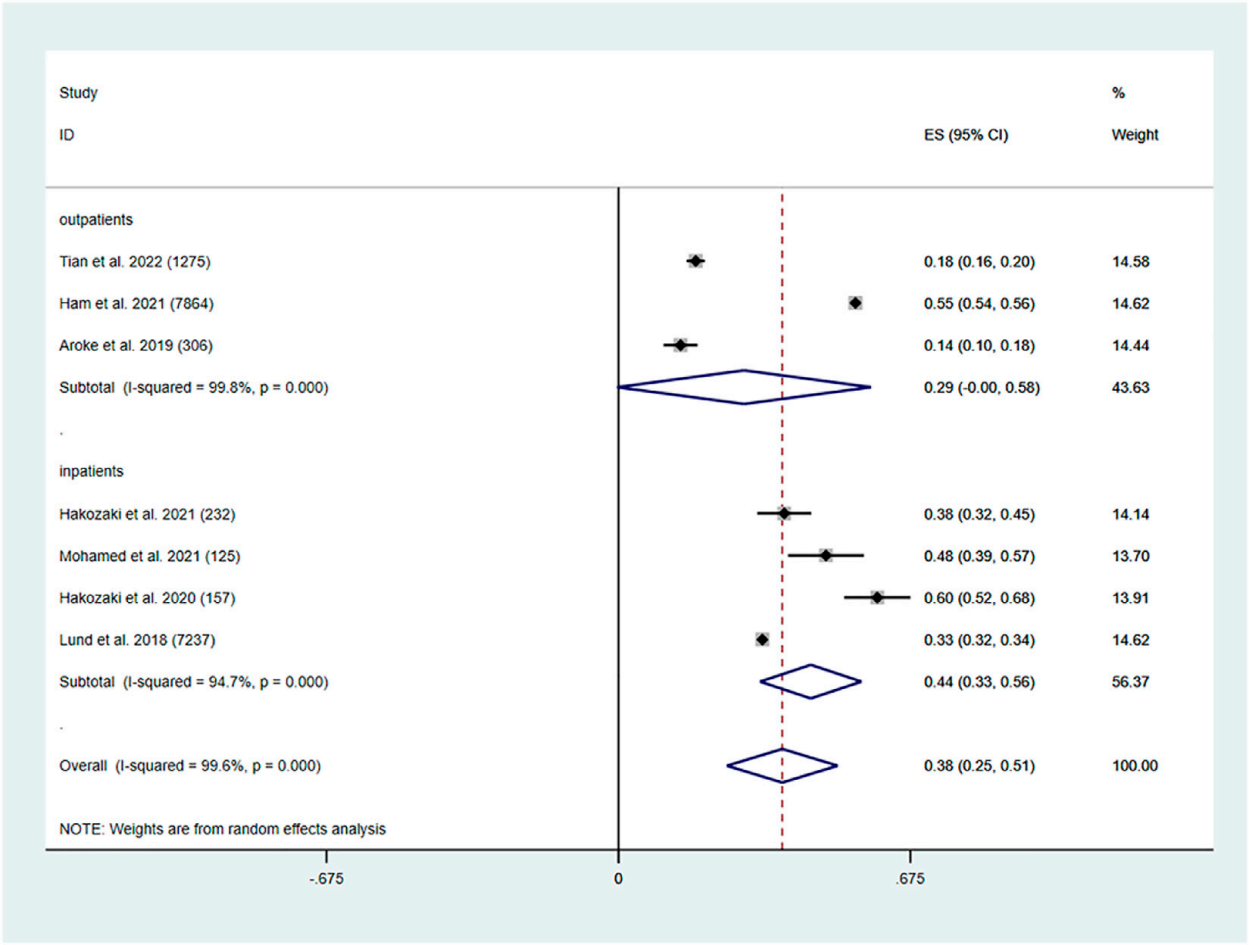
Prevalence of polypharmacy in the older lung cancer population.

Six studies, including two outpatient studies and four inpatient studies, provided information on the prevalence of polypharmacy in older lung cancer patients. The overall prevalence of polypharmacy among older lung cancer patients was 38% (95% CI: 0.25, 0.51, *p* < 0.001). In older lung cancer outpatients, the pooled prevalence of polypharmacy was 29% (95% CI: 0.00, 0.58, *p* < 0.001). Older inpatients with lung cancer had a pooled polypharmacy prevalence of 44% (95% CI: 0.33, 0.56, *p* < 0.001) (Figure 2).

**FIGURE 3:**
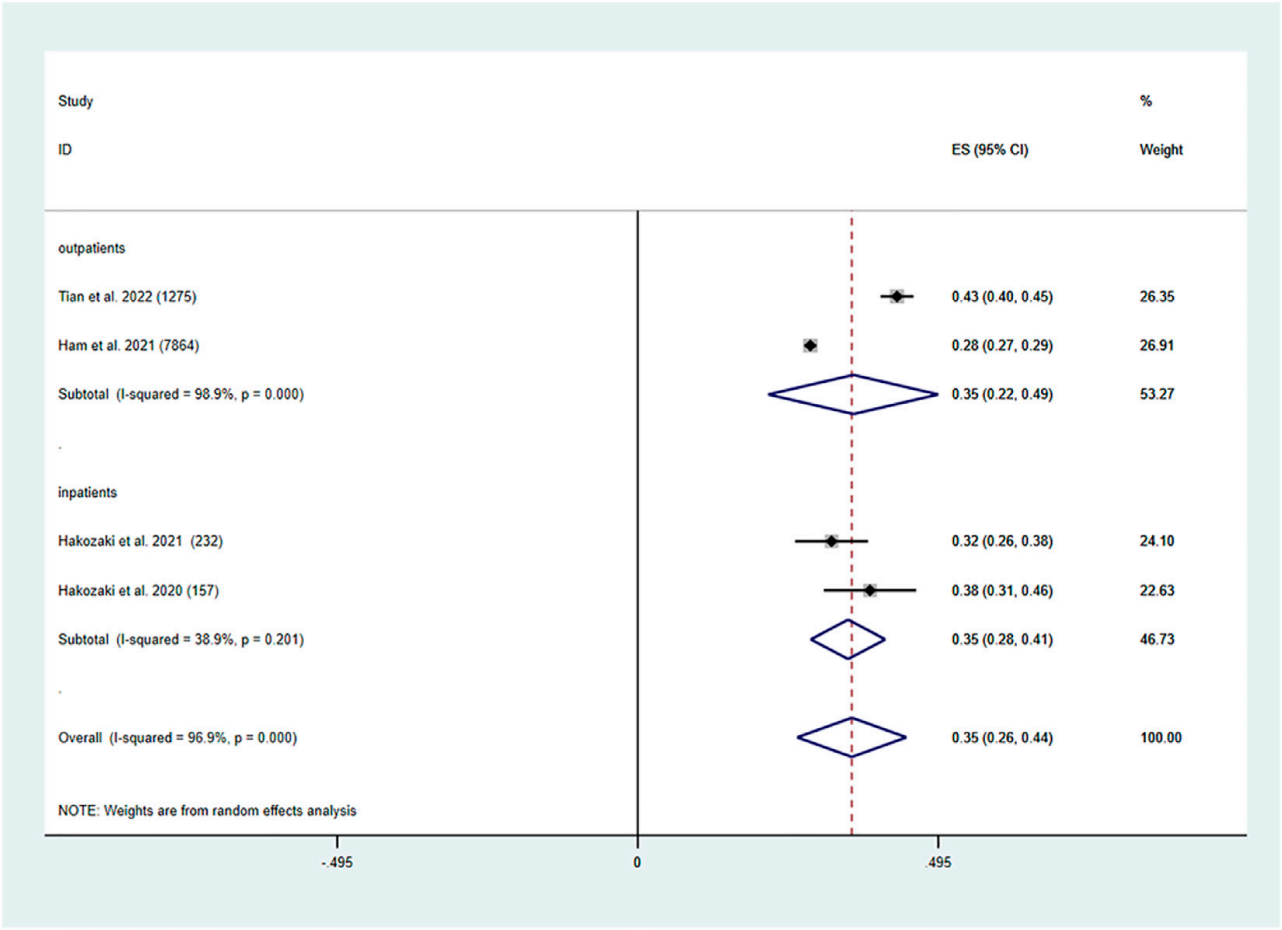
Prevalence of PIMs in the older lung cancer population.

In older lung cancer patients, the pooled prevalence of PIMs was determined to be 35% (95% CI: 0.26, 0.44, P0.001). Both older lung cancer outpatients (95% CI: 0.22, 0.49, P0.001) and inpatients had a pooled prevalence of PIMs of 35% (95% CI: 0.28, 0.41, *p* < 0.001) (Figure 3).

### Factors associated with the increased risk of polypharmacy

#### Age

Two studies focused on the correlation between the risk of polypharmacy and age change. Although the incidence of polypharmacy rose with age, there was no statistically significant difference in the risk for higher older age groups. The stratified meta-analysis revealed an increased risk (≥ 75 years old vs. 65–74 years old) of 9% polypharmacy exposure (RR: 1.09, 95% CI: 0.86,1.39, *p* = 0.486) (Table 2).

**TABLE 2:** Factors associated with increased risk of polypharmacy and PIMs.

Characteristics		Number of studies	Relative ratio, RR	95% CI	*p*
Polypharmacy					
Age	≥ 75 vs. 65–74	2	1.09	0.86, 1.39	0.486
Gender		3	0.88	0.73, 1.04	0.137
Tumor type					
	Adenocarcinoma vs. other	2	1.02	0.75, 1.37	0.92
PIMs					
Gender		2	1.04	0.99, 1.10	0.124
Tumor type					
	NSCLC vs. SCLC	2	1.04	0.96, 1.12	0.341
	SCLC vs. other	2	0.95	0.86,1.05	0.361
	NSCLC vs. other	2	0.98	0.91,1.05	0.536

NSCLC: non-small cell lung cancer; SCLC: small cell lung cancer.

#### Gender

Three studies focused on the correlation between the risk of polypharmacy and gender change. According to the meta-analysis, polypharmacy in older lung cancer patients was not associated with sex differences (RR: 0.88, 95% CI: 0.73, 1.04, *p* = 0.137) (Table 2).

#### Tumor type

Two studies focused on the correlation between the risk of polypharmacy and tumor type change according to the meta-analysis, polypharmacy in older lung cancer patients was not associated with tumor type differences (RR: 1.02, 95% CI: 0.75, 1.37, *p* = 0.92) (Table 2).

### Factors associated with the increased risk of PIMs

#### Gender

Two studies focused on the correlation between the risk of PIMs and gender change. According to the meta-analysis, PIMs in older lung cancer patients was not associated with sex differences (RR: 1.04, 95% CI: 0.99, 1.10, *p* = 0.124) (Table 2).

#### Tumor type

Two studies focused on the correlation between the risk of PIMs and tumor type change. According to the meta-analysis, PIMs in older lung cancer patients was not associated with tumor type differences (RR: 1.04, 95% CI: 0.96, 1.12, *p* = 0.341), (RR: 0.95, 95% CI: 0.86, 1.05, *p* = 0.361), (RR: 0.98, 95% CI: 0.91, 1.05, *p* = 0.536) (Table 2).

#### Different criteria

Five studies focused on the association of various criteria with the risk of PIMs. One study reported the 2019 AGS Beers criteria, two studies reported the STOPP/START criteria, one study reported the OncPal Deprescribing Guideline for patients with cancer in the palliative phase, and one study reported the 2012 AGS Beers criteria. According to the 2019 AGS Beers criteria (43%), the STOPP/START criteria (49%), the OncPal Deprescribing Guideline (28%), and the 2012 AGS Beers criteria (37%–45%), PIMs was prevalent.

## Discussion

The simultaneous use of multiple medications, including prescription pharmaceuticals, over-the-counter medicines, and nutritional supplements, is known as polypharmacy. It is generally known that polypharmacy affects life quality and increases the risk of prescription mistakes, drug‒drug interactions, and adverse drug reactions (ADRs). Although the frequency of polypharmacy varies by demographic, it has been shown that older cancer patients had polypharmacy rates of 90%.

Even though the top number of new cases in 2020 worldwide was breast cancer (226, 1419, 11.7%), the number of deaths caused by lung cancer in 2020 was the highest (179, 6144, 18%) reported by the Global Cancer Observatory 2020 (Global Cancer Observatory, 2020). The highest lung cancer incidence, mortality and 5-year prevalence worldwide were in Asia. According to Global Cancer Statistics 2020, China has the most cancer fatalities and new cases worldwide (Sung et al., 2021). At present, the number of cancer fatalities and new cases in China continues to rise, and the medical cost caused by cancer exceeds 220 billion every year. The incidence (82.8 per 10,000) and mortality (65.7 per 10,000) of lung cancer both ranked first in China according to cancer incidence and mortality in China in 2016 (Zheng et al., 2022). With increasing age, this trend is further deepened. The peak number of new cancer cases in both men and women was 60–79 years old.

In a thorough assessment of the available research and a meta-analysis, we present in our study for the first time the pooled prevalence of polypharmacy and PIMs and the risks associated with these behaviors in the older lung cancer population. We concluded that 38% of older lung cancer patients had polypharmacy overall after analyzing six trials. The prevalence of polypharmacy ranged from 17.88% to 59.87% among older lung cancer patients from four different nations. The pooled prevalence of polypharmacy of older lung cancer outpatients was 29% and that of older lung cancer inpatients was 44%. A study that focused on the use of antitumor drugs in older metastatic colorectal cancer patients reported that the polypharmacy of patients accounted for 38.7% (Yekedüz et al., 2022). A study from America found that 26% of older breast cancer patients and 29% of older colorectal cancer patients took six or more drugs every day (Lund et al., 2018). One study found the prevalence of polypharmacy in older Indian patients with lung cancer was higher than other malignancies (Vanita et al., 2021). Some recent meta-analyses reported a pooled prevalence of PIMs in older cancer patients between 19.0% and 52.0% (Mohamed et al., 2020). The AGS Beers criteria were the most common criteria used to screen for PIMs in older cancer patients. According to the study, all three cancer cohorts (breast cancer, colon cancer, and lung cancer) had identical monthly prevalence rates of any PIMs before cancer diagnosis, which ranged between 37% and 40%. While PIMs prevalence increased significantly in the first few months after a colon or lung cancer diagnosis and gradually returned to prediagnosis levels over the next 23 months, PIMs prevalence among breast cancer patients steadily decreased across the period after diagnosis (Lund et al., 2018). Compared to patients with the other two malignancies, older lung cancer patients used PIMs more frequently. According to our meta-analysis, older lung cancer patients used PIMs at a rate of 35%. One study showed that concomitant conditions are present in at least half of lung cancer patients, increasing the likelihood that PIMs will be used (Pluchart et al., 2021). In addition, 16.3% of the 31 million cancer survivors in the United States reported using several psychiatric medications. Following survivors of breast cancer (17.8%), colorectal cancer (17.8%), and other gastrointestinal cancers (16.0%), survivors of lung cancer had the greatest rate of psychotropic polypharmacy (22.5%) (Vyas et al., 2020). This was also the cause of older lung cancer patients using PIMs more frequently than those with the other two cancers.

The results of the study confirmed population aging and rising trends in the risk of polypharmacy for higher age groups. Our meta-analysis showed that neither polypharmacy nor PIMs in the older lung cancer population was substantially related to sex differences. The STOPP/START criteria were more sensitive than the other criteria, which may be because the older lung cancer patients in these two studies were inpatients, and the prevalence of PIMs in older inpatients was originally higher than that in outpatients. There were no significant findings between different tumor types of the risk of PIMs and polypharmacy in our study.

The current research shows that interventions targeting PIMs might help older people’s health outcomes and reduce medication-related damage (Mekonnen et al., 2021). PIMs usage increased polypharmacy prevalence and resulted in significant morbidity and death in older persons (Achterhof et al., 2020). Deprescribing is a recognized management strategy to minimize the prevalence of polypharmacy and PIMs. polypharmacy and PIMs (Wu et al., 2021). There is little knowledge regarding the therapeutic effectiveness of reducing polypharmacy in older patients. Deprescribing intervention in older patients who unexpectedly enter the hospital or pass away is not without risks, but its benefits and long-term viability are unclear (Rieckert et al., 2020). According to one study, older cancer patients’ polypharmacy and PIMs seemed to be evaluated in relation to pharmacist-led drug evaluation deprescribing (Nightingale et al., 2015). In comparison to interdisciplinary team interventions, other studies showed that pharmacist-led deprescribing interventions in older patients may be more successful in lowering the use of unneeded drugs (Verrue et al., 2009; Tjia et al., 2013). As a result, it is necessary to create instruments that are standardized to define the elements that constitute the appropriate and inappropriate use of polypharmacy. Furthermore, an effective intervention and management of PIMs should be conducted in older cancer patients in the future, especially in older lung cancer patients.

## Limitations

This systematic review and meta-analysis was conducted to consolidate quantitative evidence on the impact of polypharmacy and PIMs in older lung cancer patients. The study’s findings, however, have some limitations. First, there are only six studies included, so there is a certain risk of bias in the conclusions, the results might be impacted by variables including illness distribution, doctor diagnostic proficiency, and prescription practices that differ greatly between hospitals in other nations. Furthermore, most studies did not analyze the connection between polypharmacy and PIMs usage in older lung cancer patients. Third, the majority of the research that made up this systematic review and meta-analysis was carried out in North America, Europe, and Asia. As a result, the findings of this study could not accurately represent the circumstances in other nations.

## Conclusion

In older lung cancer patients, this systematic review and meta-analysis found a high prevalence of polypharmacy (38%) and PIMs usage (35%). The findings of this study indicate that older lung cancer populations should undergo effective intervention and management of PIMs in the future. The incidence of polypharmacy, PIMs, and related risk factors in older patients with various malignancies should be the subject of further investigation.

## Data Availability

The raw data supporting the conclusion of this article will be made available by the authors, without undue reservation.
